# Patisirana no Tratamento da Amiloidose Cardíaca: Bom para o Mundo e Bom para o Brasil

**DOI:** 10.36660/abc.20250335

**Published:** 2025-06-26

**Authors:** Humberto Villacorta

**Affiliations:** 1 Universidade Federal Fluminense Niterói RJ Brasil Universidade Federal Fluminense, Niterói, RJ – Brasil

**Keywords:** Amiloidose Cardíaca, Transtirretina, Silenciadores Genéticos, Tratamento

**Figura 1:**
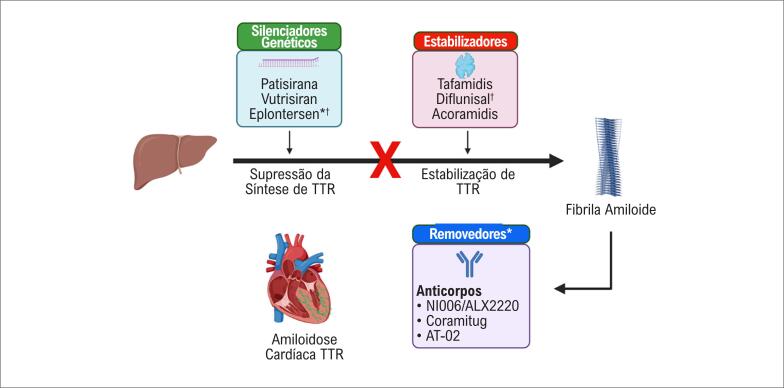
Terapia específica para amiloidose com transtirretina. TTR: transtirretina. * Ensaios clínicos em andamento em amiloidose cardíaca com TTR. † Eficaz em ensaios neuroclínicos sobre amiloidose com TTR hereditária.

Patisirana é um agente terapêutico de interferência de RNA usado no tratamento da amiloidose mediada por transtirretina (amiloidose ATTR), uma condição caracterizada pelo acúmulo de fibrilas amiloides da proteína transtirretina (TTR) mal dobradas nos tecidos.^1–4^ Conforme mostrado na Figura 1, o mecanismo de ação difere de medicamentos anteriores, como o tafamidis, que promove a estabilização da proteína TTR, em vez da inibição da síntese.^4,5^ Patisirana usa um mecanismo que envolve um pequeno RNA de interferência para atingir especificamente o RNA mensageiro (mRNA) responsável pela produção da proteína TTR no fígado. Ele ativa o complexo de silenciamento induzido por RNA, que promove a degradação do mRNA da TTR. Ao degradar o mRNA da TTR, a patisirana reduz efetivamente a síntese da proteína TTR no fígado. Isso leva à diminuição dos níveis das formas normal e mutante da TTR.^1–4^

Os efeitos da patisirana na amiloidose cardíaca foram avaliados no estudo APOLLO B, que foi um ensaio clínico de fase 3, multicêntrico, randomizado, duplo-cego e controlado por placebo envolvendo 360 pacientes diagnosticados com amiloidose ATTR hereditária ou selvagem com cardiomiopatia, com o objetivo de avaliar os efeitos do tratamento na função cardíaca e na qualidade de vida.^6^ O desfecho primário foi uma alteração em relação ao valor basal na capacidade funcional avaliada pelo teste de caminhada de 6 minutos (TC6) em 12 meses. A distância percorrida no TC6 diminuiu ao longo do tempo em ambos os grupos, mas foi atenuada no grupo patisirana (alteração média −8,15 vs −21,35 m, no grupo patisirana e placebo, respectivamente). A qualidade de vida avaliada pelo Questionário de Cardiomiopatia de Kansas City - Resumo Geral (KCCQ-OS) melhorou no grupo Patisirana e declinou no grupo placebo. Em uma análise de desfecho secundário, não foi observada diferença no desfecho combinado de morte por qualquer causa, eventos cardiovasculares ou alteração no TC6. Uma análise exploratória constatou que a patisirana atenuou o aumento dos biomarcadores cardíacos NT-proBNP e troponina I.

Nesta edição dos Arquivos Brasileiros de Cardiologia, são relatados dados da população brasileira incluída no estudo APOLLO B.^7^ Exceto por pequenas diferenças na intensidade dos efeitos, seus achados são essencialmente os mesmos do estudo multicêntrico. Os autores concluem que a eficácia e a segurança da patisirana em pacientes brasileiros com amiloidose cardíaca ATTR foram consistentes com as da população global do estudo APOLLO-B.

Esta subanálise é justificada pelo fato de que as ações dos medicamentos podem diferir de acordo com diferenças raciais e étnicas. As diferenças na resposta aos medicamentos estão principalmente relacionadas a diferenças raciais/étnicas na frequência de polimorfismos em genes que codificam enzimas metabolizadoras de medicamentos e transportadores de medicamentos. Esses polimorfismos podem influenciar a farmacocinética, as necessidades de dose e a segurança de alguns medicamentos.^8,9^ Um ponto forte do estudo é que os autores relataram dados sobre a cintilografia com pirofosfato, o que não foi relatado no estudo global. A melhora na escala de Perugini foi notável, com melhora em 11/18 (61%) pacientes no grupo patisirana e nenhuma melhora no grupo placebo (0/10 pacientes).

No entanto, este estudo apresenta uma limitação importante. A população era muito pequena, o que não permitiu uma análise estatística adequada, e os resultados, portanto, são apenas dados descritivos. Apesar disso, parabenizamos os pesquisadores brasileiros por participarem de um estudo tão importante e por fornecerem dados específicos da coorte brasileira.
