# Supplementary material for: Patisirana no Tratamento da Amiloidose Cardíaca: Bom para o Mundo e Bom para o Brasil
Source: Arq Bras Cardiol. 2025 Jun 26;122(4):e20250335. [Article in Portuguese] doi: 10.36660/abc.20250335 (PMC12269899; doi:10.36660/abc.20250335)
Supplement: Translation

# Translation: Patisiran in the Treatment of Cardiac Amyloidosis: Good for the World and Good for Brazil

**DOI:** 10.36660/abc.20250335i

**Authors:** Humberto Villacorta

**Affiliations:** 1 Niterói RJ Brazil Universidade Federal Fluminense, Niterói, RJ – Brazil

**Keywords:** Cardiac Amyloidosis, Transthyretin, Genetic Silencers, Treatment

Patisiran is an RNA interference therapeutic agent used in the treatment of transthyretin-mediated amyloidosis (ATTR amyloidosis), a condition characterized by the accumulation of misfolded transthyretin (TTR) protein amyloid fibrils in tissues.^1–4^ As shown in Figure 1, the mechanism of action differs from previous drugs such as tafamidis, which promotes the stabilization of the TTR protein, rather than the synthesis inhibition.^4,5^ Patisiran uses a mechanism involving small interfering RNA to specifically target the messenger RNA (mRNA) responsible for producing the TTR protein in the liver. It activates the RNA-induced silencing complex, which promotes the degradation of the TTR mRNA. By degrading TTR mRNA, patisiran effectively reduces the synthesis of TTR protein within the liver. This leads to decreased levels of both normal and mutant forms of TTR.^1–4^

**Figure 1:** Transthyretin amyloidosis-specific therapy. TTR: transthyretin. * Ongoing trials in TTR cardiac amyloidosis. † Effective in hereditary TTR amyloidosis neuroclinical trials.

The effects of patisiran in cardiac amyloidosis were assessed in the APOLLO B study, which was a phase 3, multicenter, randomized, double-blind, placebo-controlled trial involving 360 patients diagnosed with hereditary or wild-type ATTR amyloidosis with cardiomyopathy, aimed at assessing treatment effects on cardiac function and quality of life.^6^ The primary endpoint was a change from baseline in the functional capacity as assessed by the 6-minute walk test (6MWT) at 12 months. The walk distance in the 6MWT decreased over time in both groups, but it was attenuated in the patisiran group (mean change −8.15 vs −21.35 m, in the patisiran and placebo group, respectively). Quality of life as assessed by the Kansas City Cardiomyopathy Questionnaire-Overall Summary (KCCQ-OS) improved in the patisiran group and declined in the placebo group. In a secondary endpoint analysis, no difference was observed in a combined endpoint of death from any cause, cardiovascular events, or change in the 6MWT. An explanatory analysis found that patisiran attenuated the increase in the cardiac biomarkers NT-proBNP and troponin I.

In this issue of *Arquivos Brasileiros de Cardiologia*, data from the Brazilian population included in the APOLLO B study are reported.^7^ Except for small differences in the intensity of the effects, their findings are essentially the same as the multicenter study. The authors conclude that the efficacy and safety of patisiran in Brazilian patients with ATTR cardiac amyloidosis were consistent with those in the global APOLLO-B population.

This subanalysis is justified by the fact that drug actions may differ according to racial and ethnic differences. Differences in drug response are mainly related to racial/ethnic differences in the frequency of polymorphisms in genes encoding drug-metabolizing enzymes and drug transporters. These polymorphisms may influence the pharmacokinetics, dose requirements, and safety of some drugs.^8,9^ One strength of the study is that the authors have reported data on the pyrophosphate scintigraphy, which was not reported in the global study. The improvement on the Perugini scale was remarkable, with improvement in 11/18 (61%) patients in the patisiran group and no improvement in the placebo group (0/10 patients).

However, this study does have a major limitation. The population was too small, which did not allow for appropriate statistical analysis, and the results, therefore, are only descriptive data. In spite of that, we congratulate the Brazilian investigators for taking part in such an important study and for providing data specifically in the Brazilian cohort.
